# Synthesizing and tuning stochastic chemical reaction networks with specified behaviours

**DOI:** 10.1098/rsif.2018.0283

**Published:** 2018-08-15

**Authors:** Niall Murphy, Rasmus Petersen, Andrew Phillips, Boyan Yordanov, Neil Dalchau

**Affiliations:** 1 Biological Computation Group, Microsoft Research, Cambridge CB1 2FB, UK; 2 Sainsbury Laboratory, University of Cambridge, Bateman Street, Cambridge CB2 1LR, UK

**Keywords:** chemical reaction networks, programme synthesis, parameter optimization, chemical master equation, satisfiability modulo theories, Markov chain Monte Carlo

## Abstract

Methods from stochastic dynamical systems theory have been instrumental in understanding the behaviours of chemical reaction networks (CRNs) arising in natural systems. However, considerably less attention has been given to the inverse problem of synthesizing CRNs with a specified behaviour, which is important for the forward engineering of biological systems. Here, we present a method for generating discrete-state stochastic CRNs from functional specifications, which combines synthesis of reactions using satisfiability modulo theories and parameter optimization using Markov chain Monte Carlo. First, we identify candidate CRNs that have the *possibility* to produce correct computations for a given finite set of inputs. We then optimize the parameters of each CRN, using a combination of stochastic search techniques applied to the chemical master equation, to improve the *probability* of correct behaviour and rule out spurious solutions. In addition, we use techniques from continuous-time Markov chain theory to analyse the expected termination time for each CRN. We illustrate our approach by synthesizing CRNs for probabilistically computing majority, maximum and division, producing both known and previously unknown networks, including a novel CRN for probabilistically computing the maximum of two species. In future, synthesis techniques such as these could be used to automate the design of engineered biological circuits and chemical systems.

## 1. Introduction

A central goal of synthetic biology is to implement specified behaviours in biological systems. Chemical reaction networks (CRNs) have been used extensively to model a broad range of biological systems, gene regulatory networks [1], synthetic logic circuits [2] and molecular programs built from DNA [3].

Extensive theoretical understanding exists about the behaviour of a multitude of CRNs, and the behaviour of some networks has been exhaustively explored [4]. Methods also exist to convert CRNs into equivalent physical implementations, based on DNA strand displacement [3,5]. CRNs also provide a common language for expressing problems studied in computer science theory, including Petri nets and population protocols [6], as well as control theory and engineering [7]. The computational power of CRNs has been extensively studied [6], and it has been shown that error-free stably computing CRNs [8] compute exactly the class of semi-linear functions [9,10]. If the stability restriction is relaxed and we allow the CRN to sometimes compute the wrong answer, then it is possible to implement a register machine, meaning that CRNs are equivalent in power to Turing machines, up to a finite error [6,11,12]. Therefore, given the expressive power of CRNs and their direct correspondence to biological implementations, we sought to develop a methodology for synthesizing candidate CRNs that exhibit a specified behaviour.

Although there are procedures to generate CRNs for semi-linear predicates [10] and functions [9], primitive recursive functions [6], ordinary differential equations (ODEs)  [13] or Turing machines [6,12], the proposal of *practical* CRNs that (either stably or probabilistically) compute a given function has hitherto mostly been a manual effort. The most obvious practical consideration is that the number of components should not be too large. As an example, for synthetic gene circuits, each component must be introduced into an organism, characterized and checked for orthogonality with other engineered components, which presents a strong preference towards smaller circuits. Furthermore, each component brings a burden to the host cell resources, the extent of which is a complex relationship between the strength of the promoter and ribosome binding sites and the availability of ribosomes, amino acids, nucleoside triphosphate, etc. Accounting for such complications is difficult to automate, though some effort has been afforded recently for the construction of genetic logic circuits [14].

Two of the most common semantics for CRNs are *continuous deterministic* and *discrete stochastic*. In continuous deterministic semantics, the reactions are interpreted as a system of differential equations, on a (continuous) real-valued vector of species concentrations, which describe the evolution of the system according to mass action kinetics over time. In discrete stochastic semantics, the reactions are interpreted as state transitions in a discrete state-space composed of non-negative integer-valued molecule counts. Each state transition occurs at a time drawn from an exponential distribution that is a function of the stoichiometry of the system. The deterministic semantics approximate the mean of the stochastic semantics, but are only accurate at high molecule numbers [15]. There are also continuous stochastic semantics, which incorporate measures of variability, such as the linear noise approximation (LNA) and, more generally, moment closure techniques [16]. In this paper, we are interested in systems with low molecule numbers (40 or fewer) so the term ‘CRN’ intends the discrete stochastic semantics unless stated otherwise. Moreover, because we are interested in computation performed by a stochastic system, we consider probabilistic computation, where the correct answer is only produced with some probability. If a CRN (with rates) satisfies a specification or problem with non-zero probability we say that it (*probabilistically) computes* that specification. If a CRN satisfies a specification or problem with probability 1, we say it *stably computes* the specification.

In this study, we propose the first method to automate the synthesis of discrete stochastic CRNs, by formally specifying a desired input–output relation and automatically identifying CRNs that satisfy this behaviour with high probability.^1^ This is in contrast to other methods where CRNs are generated from other formal systems [9,12,13] or to reproduce a time series [18]. First, we identify CRNs that have the capacity to produce correct, finite computations for a given finite set of inputs. The synthesis problem is more challenging than verification, where the goal is to determine the correctness of an existing CRN [19]. We express CRN synthesis as a satisfiability modulo theory (SMT) problem, which can be addressed using solvers such as Z3 [20]. This allows us to generate a number of candidate CRNs of a given size, in terms of the numbers of reactions, species and computation lengths, that satisfy the design constraints, or to prove that no such CRN exists. However, while the existence of correct computations is guaranteed for each generated CRN, the probability of these computations occurring might be low.

To determine whether correct computations can occur with high probability, we next optimize the reaction rates of each generated CRN. To solve the optimization problem, we combine stochastic search strategies based on Markov chain Monte Carlo (MCMC) with numerical integration of the chemical master equation (CME). This part of the problem was recently addressed in [21,22], though applied only to a single input. We specifically focus on *uniform* CRNs, which have the desirable property that the number of species and reactions does not depend on the input value. We restrict our attention to bimolecular CRNs with precisely two reactants and two products in every reaction. Bimolecular CRNs (with all rates equal to 1) are equivalent to population protocols (PPs) [8] and also guarantee that mass is conserved in the system.

An alternative synthesis strategy was proposed in [18], which used a defined sketch of possible reactions and species of the desired CRN and a specification of the desired temporal behaviour as constraints for an SMT solver. In addition, the LNA was used to search for optimal (in species numbers and accuracy) CRNs and parameters. While the use of such an approximation allows for more efficient synthesis, it assumes that the stochastic mean is identical to the solution of the deterministic rate equations. As a result, many interesting systems that make use of stochasticity at low molecule numbers will not be identified. For example, the division CRN in §3.3 does not work at all when approximated with the LNA.

More generally, the application of SMT solvers together with sketching approaches, as in [18] for CRN synthesis, have also proven successful in the context of programme synthesis. For example, in [23] a syntax-guided framework was developed for the synthesis of input–output functions, Gulwani *et al*. [24] focused on the problem of synthesizing loop-free programmes from a library of components, and Bloem *et al*. [25] considered the synthesis of distributed, fault-tolerant systems. While our approach does not rely on user-specified sketches, we restrict the search space by considering only CRNs with a given number of reactions and species. Furthermore, we allow for probabilistic solutions by identifying systems that are capable of producing correct computation paths for a range of inputs, rather than requiring that all executions for all inputs satisfy the specification as in programme synthesis. A similar strategy has also been used for the synthesis of biological programmes in [26,27], where the goal is to guarantee that certain experimentally observed executions of a system can be reproduced. While Z3 is also used as the backend SMT solver in [26,27], the focus there is on qualitative models of genetic regulatory networks. By contrast, we consider stochastic CRNs as models of biochemical systems, which makes the overall approach and encoding details different and necessitates a rate optimization step in addition to the reaction network synthesis.

SMT solvers such as Z3 support rich combinations of theories that could be used to extend the approach proposed here. For example, stochastic CRNs, rather than their non-deterministic abstractions, can be encoded using the theory of reals or approximated using bit-vectors [28]. However, addressing CRN synthesis while considering probability of correctness requires reasoning over sets of paths, which makes the problem more challenging. The SMT of reals could also be used to reason about reachability in rate-independent CRNs with continuous semantics [29], as an alternative to the use of specialized ODE solvers as in [18].

In this work, our aim is to synthesize CRNs that exploit stochasticity to probabilistically compute a specified function. The target application is to suggest CRNs that might be implemented in some programmable chemistry such as DNA computing or genetic circuits. For this scenario, we chose to generate and optimize circuits that would be as accurate as possible in a predefined input range and in a predefined time period (enabling the experimenter to indicate how long they are willing to wait). Our method also has applications as a tool for theorists to explore CRNs that compute specified functions.

We first applied our approach to synthesize CRNs capable of solving majority decision-making, a problem well studied in the literature [30–36]. Our method identified known CRNs that give probabilistic solutions in optimal time [31–33] and also revealed a novel asymmetric solution (§3.1.2). Next we considered the maximum function for which the known stable CRN [9] has four reactions and (in bimolecular form) uses eight species. Our method identified a smaller CRN that to our knowledge is a novel approximation of the maximum function (see §3.2). These examples illustrate the potential for automatically determining CRNs with specified behaviour. Finally, we applied our method to Euclidean division, a non-semi-linear function for which it is believed there are no stable, stochastic CRNs. All CRNs that we enumerated automatically were unable to compute Euclidean division with high probability, though we showed that a larger circuit, compatible with our framework, is a good probabilistic solution. Overall, we show that it is possible to enumerate and optimize CRNs that probabilistically compute a variety of functions.

## 2. Methods

### 2.1. Preliminaries

A CRN is a tuple 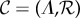, where *Λ* = {*s*_0_, …, *s*_*N*_} and 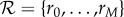 denote the finite sets of species and reactions, respectively. A CRN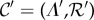, such that *Λ*′⊆*Λ* and 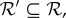 is called a *subnetwork* of 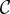. A reaction is a tuple *r* = (**r**^*r*^, **p**^*r*^, *k*^*r*^), where **r**^*r*^ and **p**^*r*^ are the reactant and product *stoichiometry* vectors (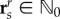 and 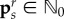 denote the stoichiometry of each species *s*∈*Λ* in **r**^*r*^ and **p**^*r*^, respectively), 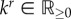 denotes the rate constant of *r* and **k** denotes the vector of all reaction rates. Given a reaction *r* = (**r**^*r*^, **p**^*r*^, *k*^*r*^), the set of reactants of *r* is {*s*∈*Λ* | **r**^*r*^_*s*_ > 0} and the set of products of *r* is {*s*∈*Λ* | **p**^*r*^_*s*_ > 0}. In this paper, we focus on the class of *bimolecular* CRNs, where 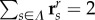 and 
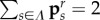
, for all reactions 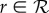.

The dynamical behaviour of bimolecular CRNs can be understood as follows. The set of all possible system states is 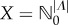, where a state 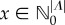 represents the number of molecules of each species. We denote the number of molecules of species *s*∈*Λ* in state *x* by *x*_*s*_. Given a reaction 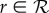 where **r**^*r*^_*s*_ = 2 for some *s*∈*Λ*, the *propensity*^2^ of *r* on *x* is *k*^*r*^_*x*_ = *k*^*r*^ · *x*_*s*_· (*x*_*s*_ − 1)/2. If, on the other hand, **r**^*r*^_*s*_ = **r**^*r*^_*s*′_ = 1 for some species *s*, *s*′, the propensity of *r* is *k*^*r*^_*x*_ = *k*^*r*^ · *x*_*s*_ · *x*_*s*′_. The time at which reaction *r* would fire, once the system enters state *x*∈*X*, is stochastic and follows an exponential distribution with a rate determined by the reaction's propensity *k*^*r*^_*x*_. Assuming that reaction *r* is the first one to fire, the state of the system is updated as *x*′_*s*_ = *x*_*s*_ − **r**^*r*^_*s*_ + **p**^*r*^_*s*_ for all *s*∈*Λ*, where *x* and *x*′ are the current and next states, respectively.

An abstraction of CRNs that preserves reachability but does not consider reaction rates or time is given by the *transition system* 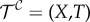, where 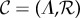 and the transition relation *T* is defined as
2.1
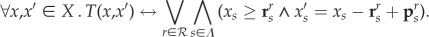


In other words, the choice between reactions from 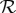 is non-deterministic but enough molecules of each reactant must be present in state *x* for the reaction to fire. The transition between states *x* and *x*′ happens when any reaction 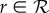 fires and the number of molecules is updated accordingly. A path *x*_0_, *x*_1_, … of 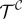 satisfies *T*(*x*_*i*_, *x*_*i*+1_) for *i* = 0, 1, … and, given an initial state *x*_0_, we call state *x*_*f*_ reachable from *x*_0_ in 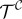 if there exists a path *x*_0_, …, *x*_*f*_.

Given a CRN 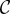, let *X*_0_ ⊆ *X* denote a finite set of initial states and *X*_*r*_ ⊆ *X* denote the set of states reachable from *X*_0_. 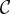 can be represented as a *continuous-time Markov chain* (CTMC) that preserves information about the transition probabilities and rates that determine the stochastic behaviour of the system and the expected execution times. We define a CTMC to be a tuple 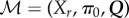, where *X*_*r*_ is a finite set of states, 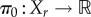 is the initial distribution of molecule copy numbers of all species, and 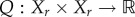 is a matrix of transition propensities. While the set of initial states is not represented explicitly, it is captured through the initial distribution, i.e. *X*_0_ = {*x*∈*X*_*r*_ | *π*_0_(*x*) > 0}. A CTMC 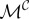 is constructed from a CRN 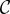 by first determining the set of reachable states, and then evaluating the propensities of each reaction. The (*i*, *j*)th entry of 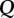, *q*_*ij*_, represents a transition from state *x*_*i*_ to state *x*_*j*_. Accordingly, *q*_*ii*_ is the remaining probability mass, equal to 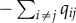. The transient probability vector *π*_*t*_ evolves according to 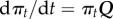, which is known as the chemical master equation (CME).

While the dynamics of the CME depend on the rate constants of the CRN (**k**), the state graph of the associated CTMC does not. Therefore, when attempting to find CRNs with specific behaviours, it is possible to separate reachability properties from dynamic behaviours, by considering changes in **k** or not. Eventually, we consider finding rate constants **k** that are optimal with respect to the desired behaviour. Following [21,37], we therefore introduce the notion of a parametric CTMC (pCTMC), which is a CTMC with transition rates parametrized by **k**. More formally, if we denote by 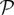 a parameter space, 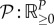, then **k** is instantiated by a parameter point 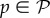. Accordingly, given a pCTMC 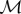 and parameter space 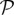, an instantiated pCTMC 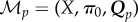 is an evaluation at point 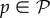.

### 2.2. Example

An example CRN 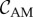 defined by
2.2*a*
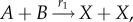

2.2*b*
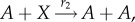

**Figure 1.**
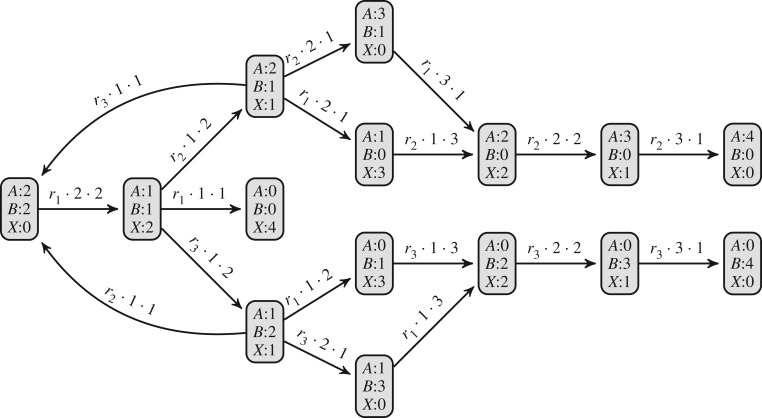
The example CRN 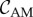 and the CTMC generated from 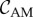 with initial state {2*A*, 2*B*}.

2.2*c*
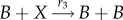
consists of the set of species {*A*, *B*, *X*}. Reaction (2.2*a*) has two reactants, *A* and *B*, two products, *X* and *X*, and occurs at rate *r*_1_. The pCTMC 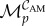 generated from the CRN 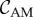 and initial state {2*A*, 2*B*} can be seen in figure 1. The corresponding transition system 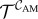 is identical to the CTMC except that the rates of the transitions are ignored. Here, the parameter point *p* is the set of reaction rates {*r*_1_, *r*_2_, *r*_3_}.

In the state graph (figure 1), each transition is obtained by applying a reaction to a source state, which identifies a target state through updating the molecule counts of the source state. The rate of the transition is obtained as the rate of the reaction multiplied by the number of ways in which the reaction can be applied. For example, from the initial state {2*A*, 2*B*} there is a single transition obtained by applying reaction (2.2*a*), which consumes one copy of species *A* and one copy of species *B*, and produces two copies of species *X*, resulting in the state {*A*, *B*, 2*X*}. This reaction has rate *r*_1_ and can be applied in four ways, since each of the two copies of species *A* can interact with each of the two copies of species *B*, resulting in a transition rate of 4*r*_1_. There exists one path between {2*A*, 2*B*} and {4*X*}, which is the sequence of states through the intermediate state {*A*, *B*, 2*X*}.

### 2.3. Problem formulation

The main problem we consider in this paper is the identification of CRNs that satisfy given properties. Specifically, we are interested in finite reachability properties, which capture a range of interesting CRN behaviours.

Let 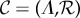 be a given CRN and 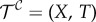 and 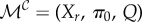 denote its transition system abstraction and CTMC representation, respectively, as discussed in §2.1. Let *ϕ* : *X* → {0, 1} denote a state predicate; for example, *ϕ*(*x*) = *x*_*s*_ > 5 specifies that there must be more than five copies of species *s* at state *x* (see appendix A for a formal definition).

In this paper, we consider *path predicates* *ψ* = (*ϕ*^0^, *ϕ*^F^), which are expressed using two state predicates that must be satisfied at the initial (*ϕ*^0^) and at some final (*ϕ*^F^) state of a path. Let *K* denote the number of steps we consider.

**Definition 2.1.**

Given a finite path *ρ* : *x*_0_ … *x*_*K*_ of 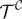 we say that *ρ* satisfies path predicate *ψ* = (*ϕ*^0^, *ϕ*^F^), denoted as 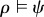, if and only if *ϕ*^0^(*x*_0_)∧*ϕ*^F^(*x*_*K*_) evaluates to true and no reactions are enabled in *x*_*K*_ (i.e. *x*_*K*_ is a terminal state).

We define the probability of *ψ*, denoted *P*_*ψ*_, using 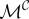 as follows. Let *X*_0_ = {*x*∈*X* | *ϕ*^0^(*x*)} denote the set of states that satisfy the initial state predicate. We initialize 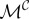 with a uniform sample from the states *x* that satisfy *ϕ*^0^, which defines *π*_0_(*x*, **k**) as a function of the rate parameters **k** as

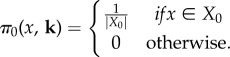
Similarly, *X*_*F*_ = {*x*∈*X* | *ϕ*^F^(*x*)} denotes the set of states satisfying the final state predicate.

**Definition 2.2.**

The probability of *ψ* is defined as

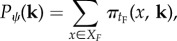
where *t*_F_ denotes the maximal time we consider and *π*_*t*_F__ is the probability vector at time *t*_F_ computed using the CME introduced in §2.1. In other words, we define *P*_*ψ*_(**k**) as the average probability of the states satisfying *ϕ*^F^ at time *t*_F_, for rate parameters **k**.

If 0 < *P*_*Ψ*_(**k**) < 1, we say the CRN *computes* or *probabilistically computes* the specification. If *P*_*Ψ*_(**k**) = 1, we say it *stably computes* the specification. By extension, we also refer to a CRN (stably or probabilistically) computing a problem where the CRN satisfies path predicates beyond the set of path predicates used in the generation phase. This is a more restrictive notion of computation than is usually used in the literature. Firstly, we require finite paths which end in a terminal state, instead of the more usual either finite or infinite paths (e.g. [8–10]). Secondly, our specifications only consider a finite set of input configurations rather than an infinite input set. We informally check the generalizability of a CRN for a specification by evaluating it on inputs beyond the initial set of path predicates.

It is important to note that definition 2.2 does not reward circuits that reach a high probability before the final time. While in principle it is possible to optimize for both speed and accuracy by, for example, defining an integral performance metric (e.g. the integral of *P*_*Ψ*_ over time), a compromise would arise between early accuracy and final accuracy. As such, the choice of *t*_F_ in the integral performance metric would implicitly control the importance of early versus final accuracy. Owing to this complication, we have decided to only consider *final accuracy* in this article. While we are still forced to choose *t*_F_ arbitrarily, we vary the rate constants over ranges that enable most circuit simulations to equilibrate by this time.

We are now in a position to formally define the problem being solved in this article.

**Problem 2.3.**

Given a finite set of path predicates 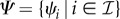, where 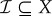 is the set of input configurations, find a bimolecular CRN 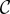 and a vector of rate parameters **k** = **k**_opt_ such that

(1) for each *ψ*_*i*_, there exists a path *ρ*_*i*_ of 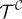, such that 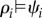, and
(2) **k**_opt_ maximizes the averaged probability of path predicates 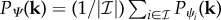 defined using 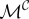.

### 2.4. Synthesis and tuning of chemical reaction networks

We solve problem 2.3 by addressing each of the two subproblems separately. First, we generate a number of CRNs that satisfy the specifications from problem 2.3(1) using an SMT-based approach (§2.4.1). The CRNs identified at that point are capable of producing a path that satisfies each path predicate, which addresses problem 2.3(1), but they might also include incorrect paths and the probability of correct computations might be low. Therefore, we tune the reaction rates of these CRNs in order to maximize the average probability (discussed in §2.4.2), which addresses problem 2.3(2).

#### 2.4.1. Satisfiability modulo theory-based synthesis

Here, we present our approach to finding a bimolecular CRN 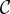 that satisfies a specification expressed as path predicates *Ψ* (problem 2.3(1)). We address this problem by encoding 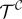 symbolically for any possible bimolecular CRN 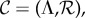 where 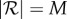 and |*Λ*| = *N* (i.e. the number of species and reactions is given), together with the specification *Ψ* for some finite number of steps *K*, as an SMT problem. We then use the SMT solver Z3 [20] to enumerate bimolecular CRNs that satisfy the specification or prove that no such CRNs exist for the given *N*, *M* and *K*. Finally, we apply an incremental procedure to search for CRNs of increasing complexity (larger *N* and *M*) or to provide more complete results by increasing *K*.

Using Z3's theory of linear integer arithmetic, we represent the stoichiometry of 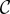 as two symbolic matrices 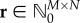 and 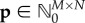 (using integer constraints to prohibit negative stoichiometries). Given a reaction 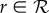 and species *s*∈*Λ*, **r**^*r*^_*s*_ and **p**^*r*^_*s*_ defined in §2.1 are now encoded as symbolic integers. We ensure that only bimolecular CRNs are considered by asserting the constraints 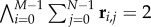 and 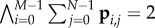. In addition, we introduce the following constraints.

— We label a subset of the species *Λ*_*I*_ ⊆ *Λ* as inputs and assert that 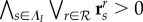 to ensure all inputs are consumed by at least one reaction.
— We label a subset of the species *Λ*_*O*_ ⊆ *Λ* as outputs and assert that 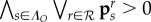 to ensure all outputs are produced by at least one reaction.
— We assert that 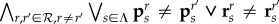 to ensure that two reactions never have the same reactants and products and, therefore, all *M* reactions are used.
— Finally, we assert that 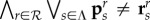 to ensure that the firing of each reaction updates the state of the system.

Following an approach inspired by bounded model checking (BMC) [38], we represent the finite path *ρ*_*i*_ = *x*^*i*^_0_, … , *x*^*i*^_*K*_ for each *ψ*_*i*_ (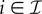, a set of initial configurations) by defining each state as a symbolic vector 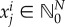 and ‘unrolling’ the transition relation of 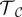 (i.e. asserting the constraint *T*(*x*^*i*^_*j*_, *x*^*i*^_*j*+1_) for each 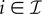 and *j* = 0 … *K* − 1). For each path predicate *ψ*_*i*_ = (*ϕ*^0^, *ϕ*^F^) and path *ρ*_*i*_, we then assert the constraint *ϕ*^0^(*x*^*i*^_0_)∧*ϕ*^F^(*x*^*i*^_*K*_)∧Terminal(*x*^*i*^_*K*_) according to definition 2.1, where 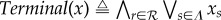, i.e. no reactions are possible due to insufficient molecules of at least one reactant.

The parameter *K* specifies the maximal trajectory length that is considered. The BMC approach is conservative, since computations that require more than *K* steps (reaction firings) to reach a state satisfying *ϕ*^F^ will not be identified. Increasing *K* leads to a more complete search, and indeed the approach becomes complete for a sufficiently large *K* determined by the diameter of a system, but also increases the computational burden.^3^ To alleviate this, we follow an approach from [19] and consider *stutter* transitions (corresponding to multiple firings of the same reaction in a single step) by using the following modified transition relation definition *T*_*st*_ (as opposed to *T* from equation (2.1)):

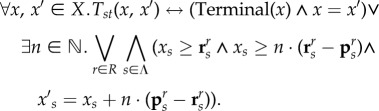
For any enabled reaction *r* (*x*_*s*_≥**r**^*r*^_*s*_), *T*_st_ allows *r* to fire up to *n* times in the stutter transition. *n* is limited by the consumption and production of the species needed for the reaction to fire (*x*_*s*_ ≥ *n* · (**r**^*r*^_*s*_ − **p**^*r*^_*s*_)). In many cases, stutter transitions dramatically reduce the required trajectory lengths (*K*_*s*_ denotes the number of stutter transitions in a trajectory), since multiple copies of the same species can react simultaneously. However, this is not restrictive, since for *n* = 1 the original definition of *T* is recovered. In addition to such stutter transitions, *T*_st_ allows self-loops at terminal states, and therefore computations that require fewer than *K*_*s*_ steps to reach a state satisfying *ϕ*^F^ can also be identified.

The encoding strategy described so far allows us to represent CRN synthesis as an SMT problem and apply an SMT solver such as Z3 [20] to produce a CRN that satisfies the specification or prove that no such CRN exists for the choice of *M*, *N* and *K* (or *K*_*s*_). More specifically, a solution CRN 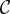 is represented through the valuation of **r** and **p**, which are extracted from the model returned by Z3.

In general, we are interested in enumerating many (or all possible) CRNs for the given class (defined by *M*, *N* and *K* or *K*_*s*_), which ensures that no valid solutions are omitted at that stage. To do so, we apply an incremental SMT-based procedure, where at each step we assert a uniqueness constraint guaranteeing that no previously discovered CRNs are generated. Given a concrete, previously generated CRN 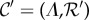 and the new symbolic CRN 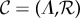 we are searching for (both of which are defined using the same species *Λ*), we define the constraint 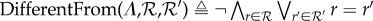, where *r* = *r*′ if and only if **r**^*r*^_*s*_ = ***r***^*r*′^_*s*_∧**p**^*r*^_*s*_ = **p**^*r*′^_*s*_ for all *s*∈*Λ*. The new CRN 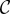 cannot simply be a permutation of the same reactions.^4^ We start by generating a solution 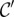 (if one exists), asserting the constraint 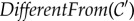, and repeating this procedure until the constraints become unsatisfiable, which corresponds to a proof that no additional CRNs exist for the given *N*, *M* and *K*.

#### 2.4.2. Tuning chemical reaction networks with parameter optimization

Here, we present our approach to optimizing the reaction rates for CRNs satisfying *Ψ*. This becomes a parameter synthesis problem over a set of pCTMCs, analogous to parameter synthesis for a single pCTMC, as studied in [21,37]. In contrast to this work, we aggregate over the multiple input combinations, as specified in problem 2.3(2).

To obtain solutions for the probability at a specified time *π*_*t*_, we used numerical integration of the CME. Specifically, we used the Visual GEC software [39] to encode the CRNs and then integrate the CME for each combination of inputs.

To solve the maximization problem, we used an MCMC method, as implemented in the Filzbach software [40]. Filzbach uses a variation of the Metropolis–Hastings (MH) algorithm to perform Bayesian parameter inference. The MH algorithm is used to approximate the posterior probability of a parameter set from a hypothesized model taking on certain values, based on a *likelihood* function that rewards parameter sets that enable the model to reproduce observation data. The probability of each parameter value is approximated by constructing a Markov chain of sampled parameter sets, such that a proposed parameter set is accepted with some probability, based on the ratio of the likelihood function evaluated at current and proposed parameter sets. For more information on MCMC methods, see [41]. MCMC methods, such as simulated annealing, have also been shown to efficiently find solutions to combinatorial optimization problems [42], taking a stochastic search approach similar to the MH algorithm. Stochastic search can provide benefits over gradient-based optimizers by maintaining a non-zero probability of making up-hill moves, protecting against getting stuck in poor local optima. To use Filzbach for optimizing CRN parameters, we use the argument of problem 2.3(2) as the likelihood function, but rescale so that a proposed parameter set will be accepted at probability 0.25 if it is 1% worse than the current parameter set. Subsequently, we generate MCMC chains with suitably many burn-in iterations and samples to obtain an approximate optimizing parameter set **k**.

## 3. Results

One of the main contributions of this paper is a method for the identification of CRNs that satisfy given properties (§2.4). The method addresses problem 2.3, allowing us to search for CRNs for which there exists a computation path that satisfies the set of path predicates (problem 2.3(1)), then optimize the rate parameters to maximize the probability that the behaviour is satisfied (problem 2.3(1)). In the following, we illustrate the method by identifying CRNs that probabilistically compute a range of specifications.

### 3.1. Probabilistic majority computation with symmetric and asymmetric chemical reaction networks

The *majority* problem (also known as the *binary consensus* problem) is one of the most analysed functions in distributed computing [36,43]. To solve the majority problem (Maj), a system must convert the smaller of two given finite populations into the larger population.

**Figure 2.**
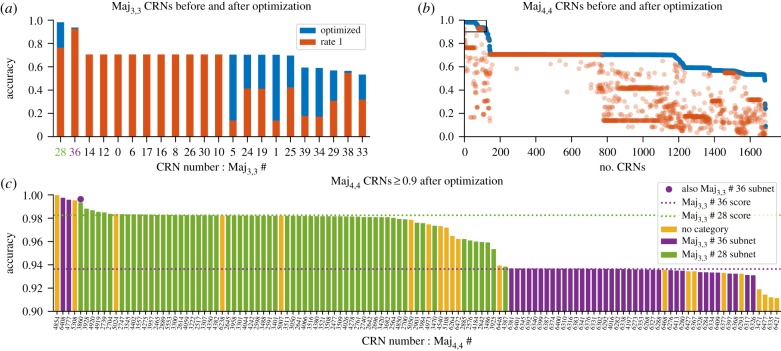
Probabilities of CRNs correctly computing the majority specification. (*a*) Results of optimizing the CRNs with three species and three reactions (found 22 CRNs) and (*b*) CRNs with four species and four reactions (found 1687 CRNs). The CRNs, unique under specification isomorphism, satisfying *Ψ*_Maj_ are ordered by their average probability after an optimization (200 burn-in, 200 samples; blue bars or circles). We also show the average probabilities before optimization (all rates equal to 1.0; red bars or circles). (*c*) Optimized score of the CRNs in the black box marked in (*b*). These CRNs are coloured according to which of the two best Maj_3,3_ CRNs it contains as a subnetwork. The coloured dotted lines mark the optimized scores of the known network Maj_3,3_ #36 (0.936) and the asymmetric network Maj_3,3_ #28 (0.982).

**Figure 3.**
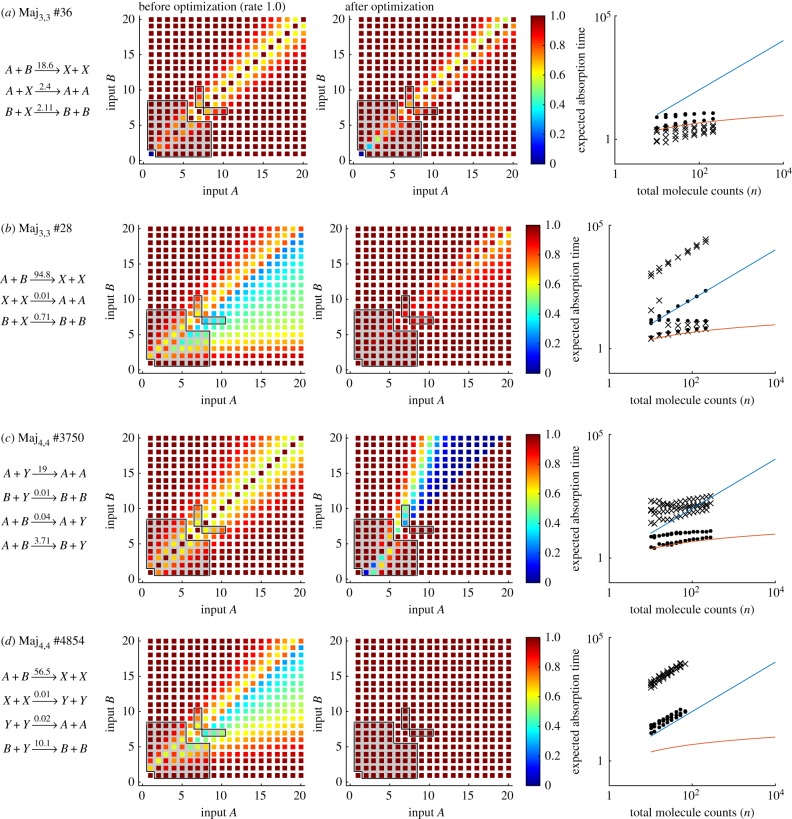
Response of probabilistic majority algorithms to varied inputs. For each input combination, specified as initial copies of species *A* and species *B*, the probability that both have the correct molecule count after 1000 time units is reported. Results are shown for a variety of networks that performed well following optimization (figure 2). The performance of each CRN is compared both before optimization (all rates equal to 1.0; left panels) and after optimization (central panels). The grey boxes show the input ranges used for both generation and optimization. The expected time until the CTMC reaches a terminal state is calculated for varying total molecule counts (*n*) (right panels). These times consider rates scaled as if occurring in a volume *n* (see §4). The completion times for three alternative initial configurations (initial copies of *A* were 10%, 60% and 90% of *n*, respectively) were calculated, illustrating minor differences in circuit completion times (cross symbols mark systems using optimized rates and filled circles mark systems using 1.0 for all rates). The red and blue lines are plots of log*n* and *n*, respectively, and give an indication of running time.

We specify the majority problem with path predicates 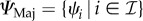, where *Λ* denotes the set of species, *ψ*_*i*_ = (*ϕ*^0^_*i*_, *ϕ*^F^_*i*_) and

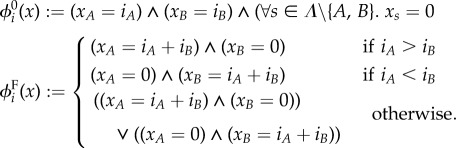
For both synthesis and rate optimization, we used the specification as instantiated with inputs *A* = *a* and *B* = *b* for all (*a*, *b*)∈*I*. That is, *Ψ*_Maj_ = {*ψ*_*i*_|(*i*∈*X*)∧(*i*_*A*_ = *a*)∧(*i*_*B*_ = *b*)∧((*a*, *b*)∈*I*)}, where *X* denotes the set of states in the CTMC and

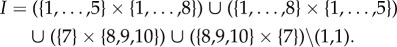
By including several input combinations, we are able to increase the penalty for incorrect computation, however each input configuration also increases the computation time for optimization, which relies on calculating the chemical master equation once per input configuration. Therefore, all input sets are selected to strike a balance between diversity of input–output behaviours and computational cost. For Maj, we specifically excluded the input combination (*a*, *b*) = (1, 1), a pathological input that precludes finding some interesting CRNs, in particular Maj_3,3_ #36 (figure 3*a*). We applied the SMT approach to identify all CRNs with two, three or four species and two, three or four reactions that satisfy *Ψ*_Maj_, using *K*_*s*_ ≤ 10 stutter steps (defined formally in §2.4.1). We then applied our optimization procedure with final time *t*_F_ = 1000 and ranked the CRNs by the optimized value of *P*_*Ψ*_Maj__ (figure 2). Overall, this revealed that there are no CRNs (with *N* ≤ 4, *M* ≤ 4 and *K*_*s*_ ≤ 10) that can solve the majority problem *stably*, that is, regardless of rates *P*_*Ψ*_Maj__ = 1 on all computation paths; however, there were several CRNs that could compute solutions with *P*_*Ψ*_Maj__ close to 1.

In the following subsections, we consider the results for *N* = *M* = 3 and *N* = *M* = 4, and analyse the majority performance of selected CRNs. We also analyse the expected time until termination, using the procedure in §4 (right-hand panels of figure 3).

#### 3.1.1. Majority computation: three species and three reactions

Out of 59 640 possible CRNs with three species and three reactions, the SMT solver found 39 CRNs where *Ψ*_Maj_ was satisfied, 22 of which were unique.^5^ Of these, two satisfied the predicate with a probability exceeding 0.9 after optimization (figure 2*a*). A score of 0.982 was reached by Maj_3,3_ #28 (figure 3*a*) and a score of 0.936 was achieved by Maj_3,3_ #36 (figure 3*b*).

**Figure 4.**
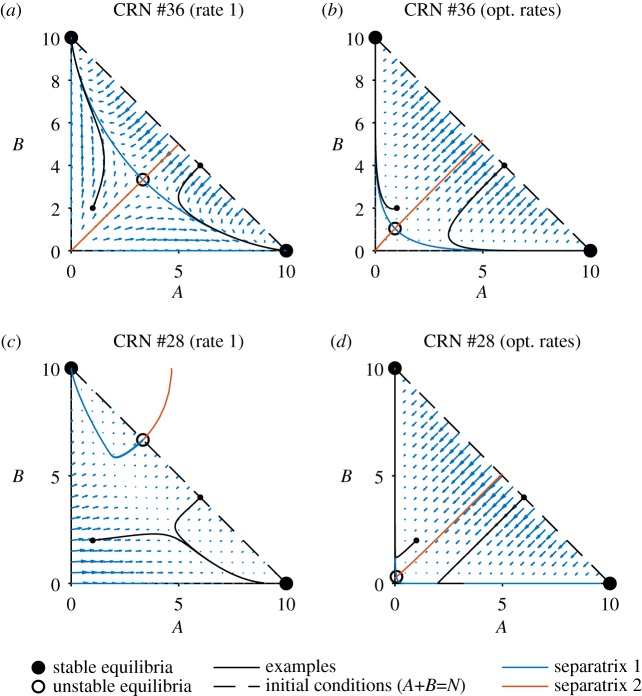
Phase portraits for CRNs computing the majority. The phase space of *A* and *B* for Maj_3,3_ #36 (figure 3*a*) and Maj_3,3_ #28 (figure 3*b*), for rate 1 and optimized rates, when interpreted as differential equations with mass action kinetics. The black dashed line represents the set of valid inputs *Ψ*_Maj_ for *N* = 10 and the black dots are the output states. The blue arrows represent the direction and magnitude of the slopes of the phase space landscape. The system has two separatrices, the blue line indicates a separatrix that represents the limit on how much of species *X* can be produced. The red separatrix represents the *A*–*B* boundary that prevents the CRN from giving incorrect solutions. The individual black lines represent paths, one a valid input of *Ψ*_Maj_ and the other an invalid input.

Maj_3,3_ #36 is the three-reaction analogue [3] of the known four-reaction *approximate majority* algorithm [31,32] (we will later identify this as Maj_4,4_ #3750). This three-reaction analogue does not satisfy the formal specification *Ψ*_Maj_ if the input configuration (*a*, *b*) = (1, 1) is included, as in this case the network terminates in the state (*A* = 0, *B* = 0, *X* = 2) and fails to make a decision. Accordingly, in figure 3*a*, we see that it scores a 0 on inputs *A* = 1, *B* = 1. The optimization of Maj_3,3_ #36 led to unequal reaction rate values, with the 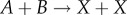 reaction being faster than the other two. To understand how this influences convergence to one of the two decision states for computations involving a large number of molecules, we prepared phase portraits of the rate equations of the continuous deterministic CRN semantics. This revealed that the optimization moved the saddle point to lower values of *A* and *B* and therefore to higher concentrations of *X* (figure 4). Analogously, analysis of the discrete stochastic CRN semantics revealed longer (and therefore slower) trajectories, and thus an accuracy–time trade-off (figure 3*a*). Nevertheless, while final accuracy is improved at the cost of slower computation, there is still a logarithmic relationship with total molecule counts.

To the best of our knowledge, the better performing Maj_3,3_ #28 (figure 3*b*) has not been analysed previously. Most likely, this is due to this network proposing an asymmetric solution to a symmetric problem, which at first appears counterintuitive. The reactions used in #28 are not isomorphic to switching the *A* and *B* species, and consequently the performance of this network with unit rates is poor and asymmetric (*P*_*Ψ*_Maj__ = 0.763; figure 3*b*, centre panel). However, optimization selects for compensatory rate values that enable surprisingly good performance in the range of inputs considered (*P*_*Ψ*_Maj__ = 0.982; figure 3*b*, right panel). Analysis of the deterministic phase portrait reveals that optimization moves the saddle point close to (*A*, *B*) = (0, 0), and produces a separatrix that almost exactly follows the line *A* = *B*. However, this accuracy comes at the cost of speed, in cases where *A* and *B* are close together the expected run times start to follow a linear trend (figure 3*b*) rather than logarithmic.

#### 3.1.2. Majority computation: four species and four reactions

We next considered CRNs with four species, to explore how many reactions are required to stably compute the majority specification. There is a known CRN (or population protocol) with four species that stably computes the majority problem with three reactions and four species [44–46]. Indeed, it has been shown that four species are necessary [35,46] to stably compute majority. However, this known CRN

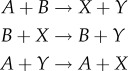
does not satisfy *Ψ*_Maj_. This is because the decision is encoded by all molecules belonging to the subset {*A*, *X*}, or all molecules belonging to the subset {*B*, *Y*} (for some applications this is more practical, e.g. [11]). As such, the decision states represent a collection of terminal states of the associated CTMC, whereas our definition involves a path predicated on a single state. However, it is easy to see that by adding two further reactions,

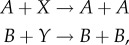
we obtain an Maj_4,5_ CRN that probabilistically computes *Ψ*_Maj_ with probability 0.946 at rate 1. In this section, we use our method to provide evidence that more than four reactions (with four species [35]) are necessary to stably compute majority (*Ψ*_Maj_).

By applying the SMT solver to four species and four reactions, we found 6486 networks (of which, 1678 are unique) satisfying *Ψ*_Maj_ out of 134 810 340 possible networks. After optimization (200 burn-in, 200 samples), we ranked the CRNs by their *P*_*Ψ*_Maj__ score (figure 2*b*).

The well-studied, optimal (in terms of reaction firings), *approximate majority* circuit [31,32], Maj_4,4_ #3750 (figure 3*c*), scored only 0.841 before optimization and 0.879 after optimization. Curiously, this was worse than the smaller CRN Maj_3,3_ #36 described above, which produced an unoptimized score of 0.926. Seemingly, optimizing for final accuracy led to asymmetric rate constants and therefore asymmetric accuracy with respect to the input values (figure 3*c*, right panel).

A large number of Maj_4,4_ networks produced an optimized score that exceeded 0.9. This is largely due to being able to append a dummy reaction to either of the high-performing Maj_3,3_ networks. Therefore, we categorized the Maj_4,4_ networks according to whether they contained Maj_3,3_ #28 or Maj_3,3_ #36 as a subnetwork (figure 2*c*). Of the Maj_4,4_ CRNs with Maj_3,3_ #36 as a subnetwork, only three (#6408, #4777, #3860; see §A.1) managed to improve on the accuracy score of that network by more than 0.5%. Of these, CRN Maj_4,4_ #3860 was an interesting case as it also has the top scoring asymmetric CRN Maj_3,3_ # 28 as a subnetwork. Other networks with Maj_3,3_ #28 as a subnetwork optimized well with most scoring over 0.98. The first (Maj_4,4_ #6408 at 0.997) and third (Maj_4,4_ #4777) best-scoring CRNs were versions of Maj_3,3_ #36 that expanded one reaction into two reactions. This increased accuracy comes at the expense of time however, as we observe a linear relationship between absorption time and total molecule counts (figure 3*d*).

We note that in our exhaustive search of the Maj_4,4_ search space there are no CRNs that stably compute majority on our input set with *K*_*s*_ ≤ 10 stutter steps.

#### 3.1.3. Numerical evaluation times

**Figure 5.**
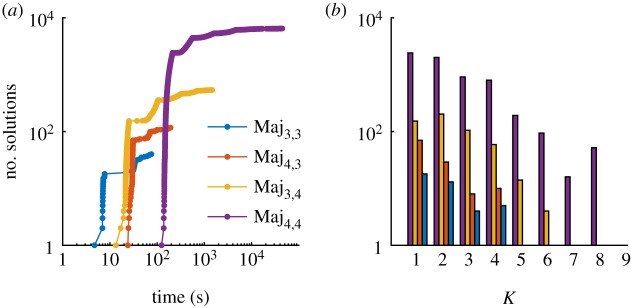
Computation times for the SMT-based synthesis of majority decision-making CRNs. (*a*) Time required to generate a number of solutions (candidate CRNs) for *Ψ*_Maj_ for *N* species and *M* reactions (denoted Maj_n,m_) for *N*, *M*∈{3, 4}^2^. (*b*) Number of solutions found as *K* (the length of considered computations with stutter transitions) increases.

The numerical evaluation times of our procedure depend on the size of the circuit (*M* and *N*), length of considered computations (*K*) and exact specification *Φ* (including the number of given path predicates). We illustrate the computation times required for the SMT-based synthesis part of our approach with the majority decision-making CRNs (figure 5).

**Figure 6.**
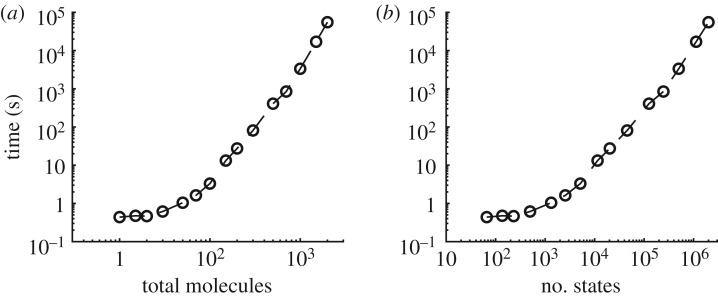
Transient probability calculation times for CRN Maj_3,3_ #36. Times indicated include the enumeration of the state space, construction of a sparse matrix, then numerical integration in the interval [0, 100/*n*], where *n* is the total molecule count. A single calculation was conducted for each value of *n*.

To determine how the numerical evaluation of the CME used in our method scales with molecular copy numbers, we first ran calculations for the established three-reaction probabilistic CRN (system Maj_3,3_ #36) for majority. As increasing the copy number decreases the simulation time interval over which there are transient dynamics, we integrated the CME over the time interval [0, 100/*n*], where *n* is the total copy number. The calculation was initialized with 0.6*n* copies of *A* and 0.4*n* copies of *B*, and all rates were set to 1. We calculated transient probabilities at 500 output points, with *n*∈[10, 1000]. This led to state spaces of varying size, up to 10^6^, with all calculations completing within 7200 s (2 h; figure 6). Smaller examples took only a few seconds.

We can approximate the total CPU run-time for parameter tuning as a function of the number of iterations of the MCMC algorithm and the number of input combinations assessed. For example, doing 200 iterations over 10 input combinations, which all have fewer than 30 total molecules (≲1 s each), suggests a tuning procedure of no more than 2000 s.

### 3.2. Computing the maximum copy number of two species

#### 3.2.1. Exact maximum computation

There is a simple bimolecular CRN to compute the minimum (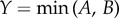) of two species,

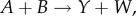
where *W* is a waste species, whereas finding the *maximum* (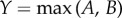) with a CRN (so far) seems to require three extra reactions. A stably computing CRN found in [9] can be converted into a bimolecular reaction by adding a ‘fuel’ species *F* (where *x*_*F*_≥*x*_*A*_ + *x*_*B*_), which uses four reactions and eight species.

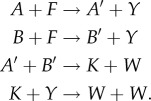


#### 3.2.2. Probabilistic maximum computation

Motivated by the stably computing circuit for maximum, we applied our technique to determine whether there exist smaller, bimolecular CRNs that probabilistically compute maximum. We specify the maximum problem using path predicates *ψ*_*i*_∈*Ψ*_Max_ for input configuration *i* (see §2.1), with *ψ*_*i*_ = (*ϕ*^0^_*i*_, *ϕ*^F^_*i*_), where

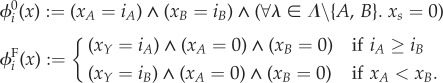
For both synthesis and rate optimization, we used the specification instantiated with input configurations *Ψ*_Max_ = {*ψ*_*i*_|(*i*∈*X*)∧(*i*_*A*_ = *a*)∧(*i*_*B*_ = *b*)∧((*a*, *b*)∈*I*)}, where

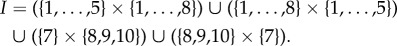


Using SMT, we identified all CRNs with three or four species and three or four reactions that satisfy *Ψ*_Max_ for *K*_*s*_ ≤ 10 stutter steps. We found no CRNs for three species with three or four reactions. For four species and three reactions, we found 128 CRNs, 66 of which were unique under specification isomorphism.

**Figure 7.**
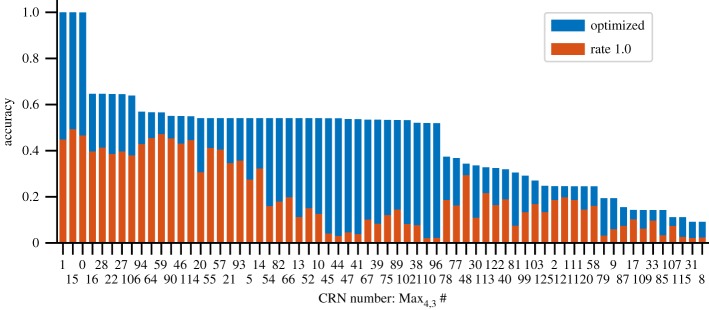
The results of optimizing the 66 CRNs unique under specification isomorphism that satisfy the path predicates *Ψ*_Max_. The top three CRNs, Max_4,3_ # 0, # 1 and # 15, all follow the same schema shown above.

We then optimized the reaction rates for final time *t*_F_ = 1000 and ranked the solutions by the value of *P*_*Ψ*_Max__ for each. Before optimization (all rates equal to 1), none of the CRNs scored above 0.5. After optimization, three CRNs reached a score of *P*_*Ψ*_Max__ = 0.999, while the fourth best scored only 0.646 (figure 7). The top three CRNs, Max_4,3_ 0, 1 and 15, are all variations of the same basic reaction schema,
3.1*a*
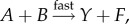

3.1*b*
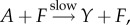

3.1*c*
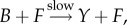
The *F*'s in the above reaction are substituted with combinations from the set {*A*, *B*, *Y* } (see §A.2). To the best of our knowledge, this is a novel CRN for probabilistically computing maximum.

**Figure 8.**
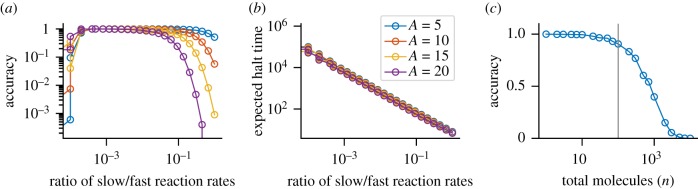
Our probabilistic circuit for maximum exhibits an accuracy–time trade-off. We parametrized the probabilistic maximum circuit Max_4,3_ #1 as: 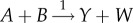, 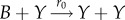, 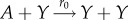 and initialized with 50 molecules in total. We then varied the slow reaction rate *r*_0_ over several orders of magnitude, altering the fast–slow ratio. (*a*) Accuracy is quantified by computing the average probability of success at time 1000. (*b*) The expected halting time was quantified using the standard halting time calculation for CTMCs (see §4). In (*a,b*) the coloured circles represent the copy number of species *A*. (*c*) The accuracy was quantified for a fixed *r*_0_ = 0.001 for 0.7*n* copies of *A* and 0.3*n* copies of *B*. The vertical line marks the *n* = 100 point. Here, accuracy is the number of stochastic simulations whose final state satisfies *Ψ*_Max_ out of 1000.

The reaction system (3.1) uses a mixture of ‘fast’ and ‘slow’ reactions. A best case computation for this algorithm relies on the input species pairing off to produce the output species (using the first *fast* reaction), before any slow reactions occur. Following this, the remainder of the more abundant species is converted to the output species, using the final two reactions, which results in the correct answer. If the final two rules fire before the first rule is completed, there is a chance that the computation will not result in the correct answer. As such, the firing of the first reaction should be as frequent as possible to reduce the chance of an incorrect computation. Conversely, the final two reactions should be as slow as possible to prevent them interfering in the first part of the algorithm. This leads to an accuracy–time trade-off (figure 8). As the ratio between the slow and fast reactions decreases, accuracy decreases (figure 8*a*), but expected hitting times increase logarithmically (figure 8*b*). Thus, it is possible to optimize the accuracy and time trade-off for a finite input range; however, beyond this range accuracy will drop rapidly to 0 (figure 8*c*) as the total number of molecules *n* increases. At the same time, the halting time increases according to *Θ*(*n*).

### 3.3. Euclidean division

So far, all of the target problems introduced in this paper are semi-linear functions, and so stably computing CRNs exist for them [9]. Division by a pre-specified constant is also a semi-linear problem, and, as such, division by 2 can be stably computed by the single-reaction CRN: 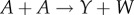. Applying our technique to division by 2 resulted in CRNs that contain the known reaction with some additional ‘helper’ reactions. These extra reactions decreased the expected time by an additive constant; however, this minor speed up came at the cost of accuracy, with maximum accuracy only when they do not fire at all. As such, we omitted graphically showing the results for the synthesis of CRNs that compute division by 2.

To challenge our CRN synthesis method, we applied our technique to compute a function that is not semi-linear, the problem of general *Euclidean division*. That is, for input quantities *a* (the *dividend*) and *b* (the *divisor*), we seek a computation of the function *a*÷*b* = ⌊^*a*^/_*b*_⌋. To formally specify the division problem, we use path predicates *ψ*_*i*_∈*Ψ*_Div_ for input configuration *i* (see §2.1) with

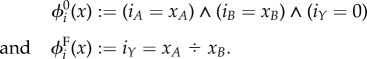


To give a diverse selection of responses, we used the specification instantiated with input configurations *Ψ*_Div_ = {*ψ*_*i*_|(*i*∈*X*)∧(*i*_*A*_ = *a*)∧(*i*_*B*_ = *b*)∧((*a*, *b*)∈*I*)}, where

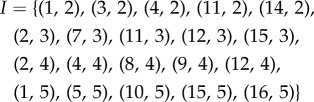
for both optimization and synthesis. This input set aims to balance the time/memory costs of optimization against the diversity of the result of the division. The list has five instances for each divisor 2, 3, 4 and 5. Without a diverse input set, we found that CRNs implementing simpler division functions could achieve high scores in optimization.

**Figure 9.**
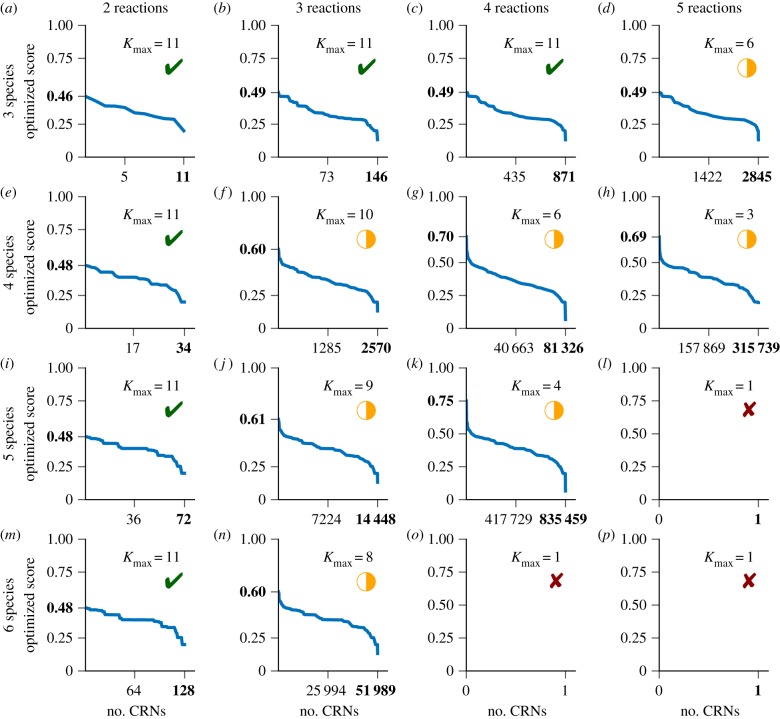
(*a–p*) Search and optimization of CRNs that probabilistically compute Euclidean division *M*÷*N*. CRNs were tested for their ability to probabilistically compute *M*÷*N* for input sets *M*∈{2, 3, 4, 5} and *N*∈{3, 4, 5, 6}. Each panel shows the number of CRNs found, the highest observed value of *P*_*Ψ*_Div__ after optimization, and the number of stutter steps *K*_*s*_ that Z3 was considering when the computation was halted after three months. *K*_max_ = 11 indicates that the computation halted and we have a complete list of CRNs up to and including *K*_*s*_ = 10.

We applied our method to search for CRNs satisfying *Ψ*_Div_ for *N* ≤ 5 species, *M* ≤ 6 reactions and *K*_*s*_ ≤ 10 stutter steps (figure 9). While the search over some combinations (*N*, *M*, *K*_*s*_) terminated, some larger combinations did not terminate within three months, and were subsequently halted. For *N* ≥ 3 and *M* ≥ 4 none of the processes terminated, illustrating a drawback in our approach to satisfying path predicates over combinatorially increasing reaction sets (6.3 × 10^9^ CRNs with four species and five reactions; 6.3 × 10^11^ CRNs with five species and five reactions; 2.6 × 10^13^ CRNs with six species and five reactions). Indeed, three processes out of four in the range *N*∈{4, 5}, *M*∈{5, 6} did not output a single CRN and never fully explored *K*_*s*_ = 1. However, for smaller bounds on *N* and *M*, we were able to generate over 10^6^ candidate CRNs that satisfied the specification encoded by *Ψ*_Div_.

**Figure 10.**
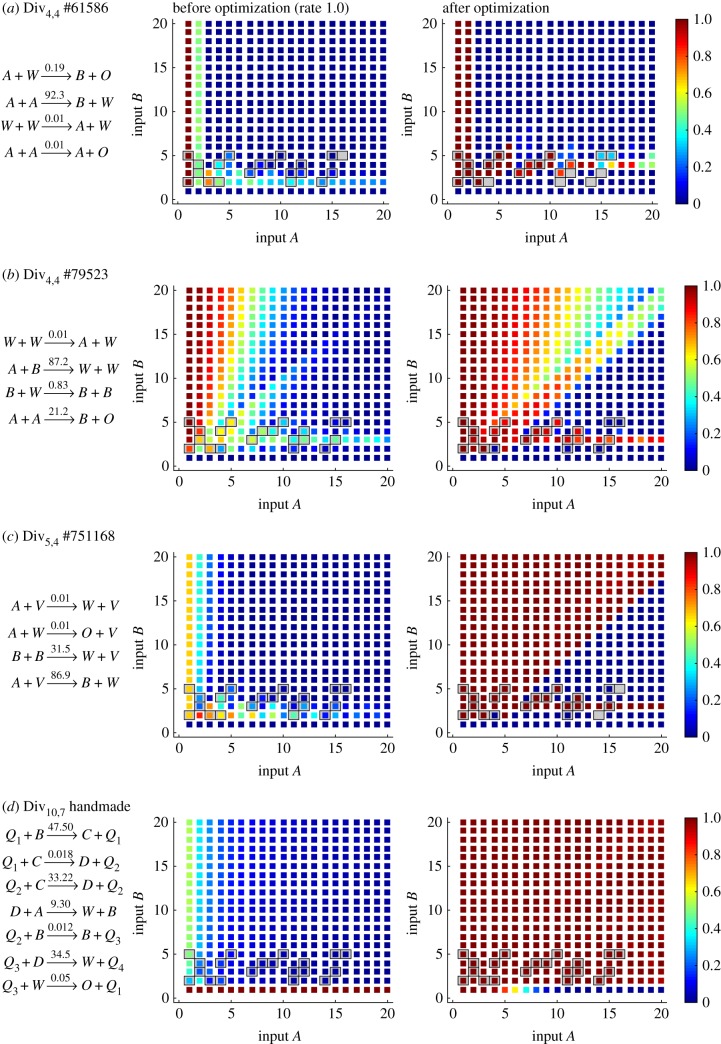
Response of selected Euclidean division algorithms to a range of inputs. For each input combination, specified as initial copies of species *A* and *B*, the probability that the molecule count of *X* is ⌊^*A*^/_*B*_⌋ after 1000 time units is reported. (*d*) is a ‘hand-made’ 10-species seven-reaction CRN that satisfies *Ψ*_Div_.

We then applied our optimization procedure with final time *t*_F_ = 1000 and ranked the CRNs by the optimized value of *P*_*Ψ*_Div__. As mentioned above, we chose the set of inputs *I* to provide a diverse set of responses that would direct synthesized networks to be general dividers by including a variety of different dividend and divisor values in the input set 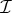. Nevertheless, we found that CRNs implementing the simpler function *f*(*x*) = ⌊*x*/3⌋ were able to achieve a score of 0.579 on *Ψ*_Div_. For example, Div_4,4_ #79523 scored 0.696 after optimization, but analysis of this CRN revealed that it produced correct outputs with high probability when the divisor was 3 (for appropriately chosen rates), but other divisors led to correct outputs with low probability (figure 10*b*). Consequently, several CRNs were optimized to use rates that make them excellent at dividing by 3. Indeed, the best 28 CRNs satisfying *Ψ*_Div_ all have their highest scores in the division by 3 row. Analogously, many CRNs instead optimized for approximating computing division by 4, for example the 28th best CRN, Div_4,4_ #61586 (score 0.655; figure 10*a*). The best CRN identified in our partial search is Div_5,4_ #751168 (figure 10), which scored 0.748 after optimization. In conclusion, in the optimization phase, it is important to have a diversity of inputs that will direct the parameter search towards a region that solves the general problem and to avoid minima where solutions score well but do not solve the problem in general. This must be balanced against the memory and time cost of stoichiometries >10.

While we were unable to automatically synthesize CRNs that compute Euclidean division with high probability, the function is known to be computable by register machines, which can be simulated probabilistically by CRNs [6,11,12]. A large CRN for Euclidean division is also sketched in [47]. Based on this, we hand-constructed a CRN with 10 species and seven reactions that takes advantage of fast (approx. 100) and slow (approx. 0.01) reaction speeds (figure 10*d*). The CRN probabilistically computes Euclidean division by counting the number of times the denominator species can be subtracted from the numerator species, and performs well, scoring *P*_*Ψ*_Div__ = 0.998 following optimization. Only when dividing by 1 does the optimized CRN perform poorly, but this is only because the computation time is limited to *t*_*f*_ = 1000, and division by 1 was not included in *Ψ*_Div_, and so rates were not optimized for this condition. The hand-made Div_10,7_ CRN satisfies *Ψ*_Div_, and makes use of *leader*^6^ molecules *Q*_1_, *Q*_2_, *Q*_3_, *Q*_4_ to control phases of the computation.

Finally, it is important to note that an alternative method to divide two numbers with CRNs is

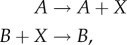
where the mean of the stationary distribution of species *X* is exactly ^*i*_*A*_^/_*i*_*B*__, where *i*_*A*_ and *i*_*B*_ are the initial values of *A* and *B* in initial configuration *i* [48]. However, because we constrain our CRN synthesis to CRNs that have reached a terminal state (see §2.4.2), the path predicates *ψ* ∈ *Ψ*_Div_ are not satisfied by this CRN. That is, we only consider CRNs that halt in the correct state and so the above division CRN is not specifiable in our scheme. While relaxing the requirement for termination is possible in principle, it is not possible to evaluate the stationary distribution in the SMT phase of our method without supplying parameter values during this phase. This would remove the benefit of filtering CRNs that do not contain a satisfying path, which we have shown to be highly stringent. The alternative would be to optimize the stationary distribution for *all* CRNs.

## 4. Discussion

In this paper, we presented a computational approach for the synthesis and parameter tuning of CRNs, given a specification of the system's correctness. We focused on the sub-class of bimolecular CRNs due to their importance as representations of various molecular algorithms and population protocols. However, our approach is more general and could also be applied directly to the synthesis of CRNs from other classes (e.g. unimolecular, trimolecular, etc.), which are defined through different stoichiometry constraints. The CRNs we synthesize can be converted into equivalent physical implementations, for example using DNA strand displacement (DSD) [3,5]. Our approach could also be applied to synthesize DSD directly, by explicitly taking into account the desired implementation strategy, such as encoding the CRN species as single-stranded DNA and the reactions as double-stranded complexes [3,5]. This could lead to simpler designs than the ones obtained through direct translation of CRNs.

We considered simple reachability properties defined in terms of predicates on the initial and final states of a computation which are sufficient to express various logical and arithmetic functions and operations. More general specifications, for example where intermediate states along computations are specified, are also currently possible within our approach but extensions to more expressive languages, such as the probabilistic temporal logics used with other methods [37], remain a direction for future work.

We first introduced our methodology for synthesizing CRNs using SMT in a conference publication [17], but this has been considerably expanded in this article. Previously, we showed that it was possible to rediscover known CRNs for majority computation, but also some unknown CRNs that are structurally asymmetric but through careful selection of their rate constants [17]. Here, we showed that the separatrices of these asymmetric majority networks approximately lie along the diagonal [*A*] = [*B*], and so trajectories starting with [*A*] > [*B*] will mostly converge to [*B*] = 0, and vice versa (figure 4). We have also shown new CRNs for majority computation that use four species, but none were able to solve the majority problem exactly (figure 2). In our first publication using this method, we also introduced attempts to find CRNs that probabilistically compute Euclidean division [17]. Here, we explored larger CRNs (figure 9), and showed that the solutions found could not provide good solutions for all divisors (figure 10). We also analysed a *hand-made* CRN comprising 10 species and seven reactions that is an excellent approximator for Euclidean division (figure 10*d*), but was beyond the computational limit for the SMT step of our method. Finally, this article introduces new results for approximating the maximum function between two species (figure 7). We showed that CRNs could make use of a combination of fast and slow reaction rates to probabilistically compute the maximum using fewer species and reactions than the previously known, stably computing, CRN [9]. Owing to the differences in rate constants, an accuracy–time trade-off arose in the computation by our CRNs (figure 8). Nevertheless, without considering such networks, biological computation schemes are likely to be more complex than might be necessary; a (bounded) loss of accuracy might be a preferable trade-off. We suggest that designing CRNs with variable rates would be difficult to achieve without automated synthesis tools.

An alternative approach to the problem of realizing arbitrary behaviour in biochemical systems is to use directed evolution [49,50]. *In silico* evolutionary search strategies might scale to larger CRNs and address the synthesis and parameter optimization sub-problems using a single, combined procedure. However, this comes at the cost of completeness, where the absence of a solution does not mean a solution does not exist. For many applications, elements of our method could be complementary to evolutionary algorithms. For example, the exact CTMC methods we use to assess the probability of correct computations in a given CRN could provide a useful fitness function for evolutionary search, compared with alternative approximate methods based on stochastic simulation. In contrast to evolutionary search strategies, our method addresses the existence and optimization sub-problems separately and uses the SMT solver and theorem prover Z3 to identify CRNs that satisfy a given specification (kinetics are ignored at this first stage). Since the results provided by Z3 are complete (up to *K* or *K*_*s*_), the termination of the procedure with no solutions is a ‘proof’ that no CRNs exist for a given specification (up to *K* or *K*_*s*_). Thus, besides providing a practical tool for the identification of CRNs with given behaviour, the completeness property means our approach could also help explore the theoretical limits of CRN computation (e.g. no CRNs with fewer than *M* species and *N* reactions that compute a given function exist). It is also important to choose a *K* (*K*_*s*_) that is large enough to accommodate the likely lengths of (stutter) trajectories needed to satisfy the specification for a given set of path predicates. However, currently large *K* or *K*_*s*_ values are costly to compute and so this limits the range of inputs we can choose for path predicates.

The fully automated generation of CRNs with discrete stochastic semantics that satisfy specified behaviours is a challenging problem and certain scalability limitations of our current method must be addressed to provide a more complete solution. Firstly, the SMT-based synthesis procedure we propose may represent large or infinite state spaces and handle systems with large molecule numbers. However, currently this method is limited to relatively small CRNs with few reactions and species, and short computation paths. Secondly, the CTMC methods we apply require an explicit representation of the state space, which must be finite (which is always the case for bimolecular CRNs initialized with a finite number of molecules). As the number of states grows exponentially in the number of species, the method is practically suitable only for systems involving relatively few species and numbers of molecules. To circumvent the need for an explicit representation of the state space, stochastic dynamical behaviour could be approximated by averaging multiple trajectories from Gillespie's stochastic simulation algorithm [51]. Instead, one could solve a synthesis problem for alternative semantics of the CRN, such as the continuous-state fluid or central-limit approximations [52], or the continuous-state deterministic rate equations. Depending on the specification, and the nature of the CRN, some of these approaches might be appropriate, but none are free of their own documented limitations. Finally, while the synthesis stage of our approach is a powerful filter on the space of all CRNs (e.g. *Ψ*_Maj_ filters out all but 6486 CRNs out of 134 810 340 possible four-species four-reaction networks, approx. 0.005%). However, as the number of reactions grows, so does the number of spurious CRNs that satisfy the specification in an uninteresting way. For example, we find many CRNs that are simple birth–death processes that can reach all possible final states. Currently, we filter these after the parameter tuning phase, which requires substantial additional computation. This indicates that additional constraints describing more accurately the structure and dynamics of ‘good’ solutions could improve the method.

We consider terminating computations by enforcing that no reactions are enabled at the state that satisfies *ϕ*^F^. Alternative strategies possible within our approach could consider reaching a fixed-point (i.e. the firing of any enabled reaction does not cause a transition to a different state), or reaching a cycle along which *ϕ*^F^ is satisfied, to guarantee that the correct output is eventually computed and remains unchanged by any subsequent reactions.

For tuning reaction rates, alternative cost functions could be used that reward solutions that are ‘nearly’ correct, e.g. using a mean-squared error. This would be most appropriate in high copy number situations, where a precise number of molecules is not integral. Our approach is focused on discrete-state stochastic semantics, and therefore most appropriate for systems operating at low copy numbers, offering an exact characterization of the probability that a specific predicate is satisfied. Our results were shown for calculations at *t*_F_ = 1000 time units, a transient probability, rather than at the stationary distribution. While the selection of *t*_F_ is subjective, it allows a circuit programmer to specify how long they are willing to wait for a computation, and provide rate constants that produce correct computations with high probability within the desired time. Circuits that reach high probability at *t* > *t*_F_ will not be rewarded more than the probability score accumulated so far by *t* = *t*_F_. However, a natural extension to the presented method would be to reward circuits that reach high probability at *t* < *t*_F_, both imposing an upper bound on time and optimizing within that range. This could be achieved by integrating our metric over the interval [0, *t*_F_].

Automating the search for CRNs that compute the solution for a specified behaviour could be beneficial to both theoretical and experimental molecular programmers. Our method can be used to show the existence or the absence of CRNs of a certain size and also suggest CRNs that can be tuned for a specific input range, and so become candidate designs for experimental construction. Prior to construction, more in-depth analysis of the candidate CRNs produced is beneficial, including parameter sensitivity/robustness analysis and bifurcation analysis (where appropriate). Future work could also incorporate notions of robustness into the proposed method, for example by using interval-based methods [37]. Nevertheless, our results provide a starting point for synthesis of CRNs with discrete-state stochastic semantics, and illustrate the potential of this approach for computing arbitrary discrete-state behaviours.

## Endnotes

1 The work presented here is an extended version of a previous conference paper [17], where we first introduced CRN synthesis using satisfiability modulo theory (SMT) solvers.

2 We assume that the reaction volume is 1 to allow for later volume scaling, e.g. 3 is the propensity for a reaction volume equal to 4.

3 Bimolecular-only CRNs are mass conserving and so their state space in guaranteed to be finite.

4 At present, our uniqueness constraint does not consider other CRN isomorphisms but certain species symmetries are broken by the specification *Ψ*.

5 In this paper, we develop the notion of ‘specification isomorphism’ to define the uniqueness of CRNs. Specification isomorphism is defined formally in §4 and is required here due to the equivalence of certain chemical species (e.g. inputs, outputs) as part of chemical computations.

6 A leader molecule is one that only takes on values 0 or 1, and can be used as a digital switch for enabling/disabling conversions of other molecules through bimolecular reactions.

## Data accessibility

All the data presented in this paper are available at doi:10.5281/zenodo.1312275 [53].

## Appendix A. State predicates

A state predicate *ϕ* is constructed using

## Appendix B. Calculating expected time

To evaluate the temporal performance of an algorithm encoded as a CRN , we make use of well-established Markov chain theory to obtain the expected time until a terminal state is reached. This is an exact measure of the expected running time for a given pCTMC with inputs , as opposed to using the mean of many stochastic simulations [11].

Let *A* ⊆ *X*_*r*_ be the absorbing states of a pCTMC  and let *τ*^*A*^ be a vector of expected hitting times, corresponding to the expected time of transitioning from a state *x*∈*X*_*r*_ to *A*. Then *τ*^*A*^ can be evaluated as the solution to the equations ([54], p. 113)

Numerical solutions can be obtained by forming a matrix *W* where the rows and columns of  corresponding to the terminal states (*A*) have been removed. Then, *τ*^*A*^ is the solution to , where  is the vector of 1's. Numerical solutions can be obtained using Gaussian elimination.

Note that the time complexity analysis of CRNs typically assumes a volume *n* equal to the maximum number of molecules in the system at any time [9]. This volume can be included by dividing each propensity by *n* before calculating expected time (see §2.1). In the case of bimolecular CRNs, this is equivalent to multiplying *τ*^*A*^ by *n*. In this work, all estimated time measures are scaled by the appropriate *n* volume.

*Parallel time* [11] is a another common measure of time complexity in the literature that may be applied to CRNs. For CRNs where all propensities are 1.0, then estimated time is identical to parallel time.

## Appendix C. Specification isomorphisms

A given CRN may be associated with a *specification* of some performed or intended computation. For example, *O* = *A* + *B* specifies that some CRN involving species *A*, *B* and *O* performs addition (specifications will be introduced formally in the following section). The addition function is commutative, which induces equivalence classes on the set of species, where {*A*, *B*} is the class of inputs and {*O*} is the class of outputs. Besides input and output species, equivalence classes could represent fuel species (e.g. species present at the beginning of a computation that are consumed during executions), waste species (species that might be absent initially but accumulate during executions) or other species roles induced by the semantics of the specification. We say two CRNs are *specification isomorphic* if they are identical under permutations of species labels within each of the equivalence classes induced by the specification (e.g. inputs, outputs, wastes, fuels). For example, the bimolecular CRN ,  is specification isomorphic under permutations of the input equivalence class {*A*, *B*}, output equivalence class {*O*} and waste {*W*}. However, it is not specification isomorphic under permutations of species in different equivalence classes such as *O* and *W* or *A* and *O*.

## Appendix D. Chemical reaction network index

**D.1. Majority**

**Table:**

CRN #	name	rate 1 score	opt. score	reactions
Maj_3,3_ #28	asymmetric (figure 3*b*)	0.764	0.982	
Maj_3,3_ #36	three-reaction AM [3] (figure 3*a*)	0.923	0.936	
Maj_4,4_ #3750	AM [31] (figure 3*c*)	0.841	0.879	
Maj_4,4_ #3860	combination of Maj_3,3_ #28 and Maj_3,3_ #36	0.828	0.993	
Maj_4,4_ #4777	third best	0.882	0.996	
Maj_4,4_ #6408	second best	0.926	0.997	
Maj_4,4_ #4854	first best (figure 3*d*)	0.831	0.999	

**D.2. Maximum**

**Table:**

CRN #	name	rate 1 score	opt. score	reactions
MAX_4,3_ #0	third best	0.465	0.99963	
MAX_4,3_ #1	first best	0.448	0.99976	
MAX_4,3_ #15	second best	0.493	0.99974	

**D.3. Euclidean division**

**Table:**

CRN #	name	rate 1 score	opt. score	reactions
Div_4,4_ #61586	approximator of division by 4 (figure 10*a*)	0.307	0.655	
Div_4,4_ #79523	approximator of division by 3 (figure 10*b*)	0.463	0.696	
Div_5,4_ #751168	the best identified (division by 3) (figure 10*c*)	0.272	0.748	
